# Assessment of Major Adverse Cardiovascular Event Across Diverse Racial Groups of Psoriasis Patients on Biologic Therapy: A Retrospective Cohort Study

**DOI:** 10.1111/1346-8138.70396

**Published:** 2026-07-26

**Authors:** Chunghwan Ro, Ana Ormaza Vera, Waleed Adawi, Clinton W. Enos

**Affiliations:** ^1^ Department of Dermatology Eastern Virginia Medical School, Macon & Joan Brock Virginia Health Sciences at Old Dominion University Norfolk Virginia USA

**Keywords:** biologics, cardiovascular, comparative effectiveness, interleukin 12/23 inhibitor, interleukin 17 inhibitor, interleukin 23 inhibitor, psoriasis, tnf inhibitor

## Abstract

Psoriasis is associated with an increased risk of major adverse cardiovascular events (MACE), and biologic therapy may reduce this risk by targeting systemic inflammation. However, limited data exist on whether long term MACE risk differs by biologic class or across racial groups. We conducted a retrospective cohort study to assess the risk of primary MACE in psoriasis patients receiving biological therapies, stratified by biological class, compared with biologic‐naïve psoriasis patients. Whether MACE risk among biologic‐treated patients varied by race was further examined. After 1:1 propensity score matching, 36 330 biologic‐treated patients were compared with 36 330 non‐biologic‐treated patients. Incident MACE was assessed over 10 years. Class‐specific analyses evaluated mutually exclusive biologic exposure groups, including TNF inhibitors (TNFi), IL‐17 inhibitors (IL‐17i), IL‐12/23 inhibitors (IL‐12/23i), and IL‐23 inhibitors (IL‐23i), each compared with an independently matched nonbiologic psoriasis comparator cohort. Race stratified analyses were restricted to biologic‐treated patients, with White patients serving as the reference group. Psoriasis patients treated with biologics exhibited a 37% lower hazard of MACE compared with non‐biologic users over the 10‐year follow‐up period [2160 vs. 3294; HR 0.63 (0.60–0.67)]. Class‐specific analyses revealed reductions in MACE risk for TNFi [1403 vs. 1844; HR: 0.74 (0.69–0.80)], IL‐17i [285 vs. 450; HR: 0.61 (0.53–0.72)], IL‐12/23i [280 vs. 374; HR: 0.73 (0.62–0.86)], and IL‐23i [238 vs. 363; HR: 0.64 (0.54–0.76)]. Race stratified analyses showed broadly similar MACE risk across racial groups; although, patients identifying as Non‐White treated with TNFi did experience a reduced MACE risk compared to White patients [127 vs. 162; HR: 0.79 (0.62–0.99)]. This isolated finding should be interpreted cautiously given multiple race stratified comparisons. Overall, biologic therapy is associated with lower 10‐year MACE risk among patients with psoriasis, with findings observed across biologic classes and largely consistent across racial strata.

## 1 Introduction

Psoriasis is a chronic inflammatory skin disease affecting approximately 2%–3% of the global population [1]. Patients with psoriasis are at an increased risk of cardiovascular disease [2] and are more likely to experience major adverse cardiovascular events [3] (MACE) beyond that attributable to measured cardiovascular risk factors [4]. This heightened risk is attributed in part to systemic inflammation [5], a key pathogenic feature of psoriasis that contributes to both the cutaneous manifestations and the increased cardiovascular burden seen in these patients.

The introduction of biologic therapies has significantly advanced the management of moderate‐to‐severe psoriasis. These targeted agents are highly effective in alleviating psoriasis symptoms and improving quality of life [6]. In addition to their dermatologic benefits, biologics reduce disease‐specific systemic inflammation [7], which may lower the risk of MACE in patients with psoriasis.

Observational studies suggest that patients receiving systemic therapies, including tumor necrosis factor‐alpha inhibitors (TNFi) and methotrexate, may have a reduced risk of MACE compared to untreated patients, although these findings are tempered by potential biases, such as the healthy user effect [8]. Mechanistic studies further demonstrate that biologic therapies may decrease vascular inflammation, coronary plaque burden, and biomarkers such as C‐reactive protein (CRP) and IL‐6, which are implicated in cardiovascular risk [9, 10, 11]. Despite these promising findings, the translation of these biomarker‐based improvements into tangible cardiovascular benefits remains uncertain [12].

While previous randomized controlled trials and clinical trial studies generally suggest that biologic therapies are not associated with an increased risk of MACE in psoriasis patients [13, 14, 15], these studies were primarily designed to evaluate dermatologic efficacy and overall treatment safety rather than long term cardiovascular outcomes. The applicability of these findings to real‐world settings remains limited due to strict inclusion criteria, shorter follow up periods, and limited sample diversity, as clinical trials often underrepresent minority populations. Although several observational studies [16, 17, 18] have evaluated cardiovascular outcomes among biologic‐treated psoriasis patients, prior work has varied in the selection of a control group, follow up duration, biologic exposure definitions, adjustment for confounding variables, and inclusion of race stratified analyses. Therefore, gaps remain regarding long term MACE risk regarding mutually exclusive biologic class exposure groups and whether these associations are preserved across racial strata. Given the paucity of research examining potential differences in cardiovascular outcomes across diverse racial groups and the well‐documented racial disparities in healthcare outcomes, it is imperative to determine whether these disparities affect the cardiovascular outcomes in the usage of biologics in psoriasis treatment [19, 20].

To address these gaps, we investigate the risk of primary MACE in psoriasis patients receiving biological therapies, stratified by biological class, compared with biologic‐naïve psoriasis patients. We further examined whether MACE risk among biologic‐treated patients varied by race. We hypothesize that biologic therapy will be associated with a reduced risk of primary MACE compared with no biologic therapy, and that this association will be broadly consistent across different racial groups.

## 2 Methods

### 2.1 Data Source

This retrospective cohort study utilized de‐identified data from TriNetX [21] from 2015 to 2025. TriNetX is a global research platform that provides real‐time access to data from a range of healthcare organizations (HCOs), including hospitals, outpatient clinics, academic medical centers, and community hospitals. The data included demographic information, diagnoses (documented according to the International Classification of Diseases, Tenth Revision, Clinical Modification [ICD‐10‐CM]), procedures (recorded using either the International Classification of Diseases, Tenth Revision, Procedure Coding System [ICD‐10‐PCS] or Current Procedural Terminology [CPT]), medications, and laboratory test results.

### 2.2 Study Population

We identified adult patients aged 18–89 years diagnosed with psoriasis. The study population was divided into two cohorts: patients receiving biologic therapies and a control group not treated with biologics. Patients with a prior history of MACE were excluded from the analysis. The biologic therapies were categorized into four classes for the analysis: tumor necrosis factor‐alpha inhibitors (adalimumab, etanercept, infliximab, certolizumab pegol) (TNFi), IL‐17 inhibitors (secukinumab, ixekizumab, brodalumab) (IL‐17i), IL‐23 inhibitors (guselkumab, tildrakizumab, risankizumab) (IL‐23i), and IL‐12/23 inhibitors (ustekinumab) (IL‐12/23i). To reduce the confounding impact of multiple therapeutic regimens, class‐specific analyses used mutually exclusive biologic‐class cohorts. Individuals in any given biologic class were excluded if they had any record of exposure to another biologic class at any point in the available TriNetX record, either before or after index. For example, participants in the IL‐17i group could not have ever received IL‐23i, IL‐12/23i, or TNFi. Accordingly, patients with cross‐class biologic switching were excluded from class‐specific analyses, whereas patients whose exposure remained within a single biologic class were retained.

### 2.3 Covariates and Propensity Score Matching

1:1 propensity score matching was utilized to balance baseline characteristics between the biologic and control cohorts, thereby reducing potential confounding biases (Table 1). Propensity score matching was performed separately for each analytic comparison. First, the overall biologic cohort was matched 1:1 to a psoriasis control cohort without biologic exposure. Second, for class‐specific analyses, each mutually exclusive biologic class (TNFi, IL‐17i, IL‐23i, and IL‐12/23i) was compared with its own independently constructed and independently matched biologic‐naïve control cohort drawn from psoriasis patients without biologic exposure. Thus, matched control cohorts shown for class‐specific analyses were generated separately for each biologic class and were not identical across comparisons. Third, race‐stratified analyses were restricted to biologic‐treated patients. Because White patients comprised more than 70% of the matched cohort, White race served as the reference group for race‐stratified comparisons. A sequential framework was used: patients whose race was recorded as other than White (pooled Non‐White) were first compared with White patients to evaluate whether the association between biologic therapy and MACE was consistent outside the majority reference group. We then performed additional race‐specific comparisons for Black versus White and Asian versus White patients, the largest individually analyzable racial groups in the dataset. Within each biologic grouping, pooled Non‐White, Black, and Asian cohorts were each separately matched 1:1 to White patients receiving the same biologic grouping. Patients categorized as Unknown Race were not included in race‐stratified comparisons because race information was unavailable in the medical record.

The covariates used in the matching process included demographic factors such as age, gender, and race (White, Black or African American, Asian, American Indian or Alaska Native, Native Hawaiian or Other Pacific Islander, Other Race, and Unknown Race), along with ethnicity. In TriNetX, Unknown Race indicates that race information was not available in the patient record. Clinical comorbidities considered were alcohol‐related disorders, overweight and obesity, nicotine dependence, hypertensive diseases, type 2 diabetes mellitus, and disorders of lipoprotein metabolism and other lipidemia. Medication usage included methotrexate, apremilast, cyclosporine, azathioprine, mycophenolate mofetil, cardiovascular medications, acitretin, and blood glucose regulation agents. Additionally, laboratory measurements such as Body Mass Index (BMI) were included to provide a comprehensive baseline assessment (Table 1). The ICD‐10‐CM codes are listed in Table 2.

**TABLE 1:** Baseline characteristics of psoriasis patients with and without biologic therapies.

Characteristics	Crude		Std diff.	Adjusted		Std diff.
	Psoriasis‐biologic (*n* = 43 930)	Psoriasis‐no biologic (*n* = 195 723)		Psoriasis‐biologic (*n* = 36 330)	Psoriasis‐no biologic (*n* = 36 330)	
Age at index, mean (SD), y	47.8 (15)	50.6 (16.5)	0.19	47.7 (15.4)	48 (16)	0.02
Gender, No. (%)						
Male	18 215 (41.5)	77 210 (39.4)	0.04	15 269 (42.0)	15 221 (41.9)	0.00
Female	23 544 (53.6)	105 455 (53.9)	0.01	19 228 (52.9)	19 396 (53.4)	0.01
Unknown gender	2171 (4.9)	13 058 (6.7)	0.07	1833 (5.1)	1713 (4.7)	0.02
Race, No. (%)						
White	32 432 (73.8)	137 232 (70.1)	0.08	26 710 (73.5)	27 371 (75.3)	0.04
Black or African American	2168 (4.9)	9410 (4.8)	0.01	1814 (5.0)	1740 (4.8)	0.01
Asian	1649 (3.8)	9987 (5.1)	0.07	1343 (3.7)	1192 (3.3)	0.02
Unknown race	5600 (12.7)	31 212 (16.0)	0.09	4743 (13.0)	4471 (12.3)	0.02
Other race	1779 (4.1)	6617 (3.4)	0.04	1475 (4.1)	1359 (3.7)	0.02
American Indian or Alaska Native	172 (0.4)	577 (0.3)	0.02	138 (0.4)	93 (0.3)	0.02
Native Hawaiian or Other Pacific Islander	130 (0.3)	688 (0.3)	0.01	107 (0.3)	104 (0.3)	0.00
Ethnicity, No. (%)						
Not Hispanic or Latino	33 094 (75.3)	143 259 (73.2)	0.05	27 179 (74.8)	27 676 (76.2)	0.03
Hispanic or Latino	2812 (6.4)	11 959 (6.1)	0.01	2326 (6.4)	2118 (5.8)	0.02
Unknown Ethnicity	8024 (18.3)	40 505 (20.7)	0.06	6825 (18.8)	6536 (18.0)	0.02
Diagnosis, No. (%)						
Hypertensive diseases	11 505 (26.189)	47 202 (24.117)	0.05	9168 (25.2)	8673 (23.9)	0.03
Disorders of lipoprotein metabolism and other lipidemias	10 352 (23.6)	45 479 (23.2)	0.01	8245 (22.7)	7700 (21.2)	0.04
Overweight and obesity	8453 (19.2)	26 233 (13.4)	0.16	6477 (17.8)	6094 (16.8)	0.03
Type 2 diabetes mellitus	4791 (10.9)	19 011 (9.7)	0.04	3783 (10.4)	3480 (9.6)	0.03
Nicotine dependence	4768 (10.9)	16 080 (8.2)	0.09	3723 (10.3)	3454 (9.5)	0.02
Alcohol related disorders	1504 (3.4)	5047 (2.6)	0.05	1238 (3.4)	1085 (3.0)	0.02
Medication, No. (%)						
Methotrexate	9884 (22.5)	6077 (3.1)	0.61	4606 (12.7)	5028 (13.8)	0.03
Apremilast	4541 (10.3)	1202 (0.6)	0.44	1294 (3.6)	1200 (3.3)	0.01
Cyclosporine	1230 (2.8)	1470 (0.8)	0.16	761 (2.1)	726 (2.0)	0.01
Acitretin	982 (2.2)	769 (0.4)	0.16	485 (1.3)	441 (1.2)	0.01
Azathioprine	426 (1.0)	748 (0.4)	0.07	307 (0.9)	288 (0.8)	0.01
Mycophenolate mofetil	199 (0.5)	682 (0.3)	0.02	143 (0.4)	186 (0.5)	0.02
Cardiovascular Medications^a^	20 581 (46.9)	74 609 (38.1)	0.18	16 063 (44.2)	15 971 (44.0)	0.01
Blood Glucose Regulation Agents^b^	7538 (17.2)	25 731 (13.1)	0.11	5851 (16.1)	5629 (15.5)	0.02

^a^ Veterans Affairs (VA) class code CV000.

^b^ VA class code HS500.

**TABLE 2:** ICD‐10 CM codes utilized within TriNetX.

ICD‐10 CM codes	Descriptor
L40	Psoriasis
I10‐I1A	Hypertensive diseases
E78	Disorders of lipoprotein metabolism and other lipidemias
E66	Overweight and obesity
E11	Type 2 diabetes mellitus
E66	Overweight and obesity
F10	Alcohol related disorders
I20‐I25^a^	Ischemic heart diseases
I46^a^	Cardiac arrest
I63^a^	Cerebral infarction
I50^a^	Heart failure

Abbreviation: ICD‐10 CM = International Classification of Diseases 10th Revision Clinical Modification.

^a^ Major adverse cardiovascular event (MACE) component.

### 2.4 Outcome Assessment

The primary outcome was the incidence of major adverse cardiovascular events (MACE), which were defined as ischemic heart diseases (ICD‐10‐CM codes I20–I25), cardiac arrest (I46), heart failure (I50), and cerebral infarction (I63). The follow‐up period for each patient was standardized to a 10‐year window from the index date, defined as the first exposure to biologic therapy for the treated cohort and a corresponding matched date for the control cohort. The time on treatment for each biologic class was calculated to account for potential differences in drug exposure duration. To assess the impact of race on MACE outcomes, analyses were stratified by race, with the White population serving as the reference group.

### 2.5 Statistical Analysis

Propensity score matching ensured an even distribution of confounding variables across the study groups. Matching was performed separately for the overall biologic versus no biologic comparison, for each class‐specific biologic versus biologic‐naïve comparison, and for each race‐stratified comparison within biologic‐treated cohorts. Propensity score matching was performed through a 1:1 matching with a nearest‐neighbor greedy algorithm and a caliper set at 0.25 times the standard deviation. All study variables were reported as means with standard deviations (SDs) for continuous variables and as frequencies with corresponding percentages for dichotomous variables. Comparisons involving continuous variables were conducted using Student's *t*‐test, while Pearson's Chi‐squared test was applied for dichotomous variables. Cox proportional hazards models were applied to estimate hazard ratios (HRs) and 95% confidence intervals (CIs) for the risk of MACE associated with biologic therapies [22, 23]. Schoenfeld residuals analysis was employed to assess the proportional hazards assumption. Statistical significance was defined as a two‐tailed *p*‐value < 0.05. All analyses were performed using the TriNetX analytic suite.

## 3 Results

### 3.1 Psoriasis Patients Treated With a Biologic Had a Lower MACE Risk Compared to Those Not Treated With a Biologic

Prior to propensity score matching, 239 653 psoriasis patients were identified, of whom 43 930 received biologic therapy, while 195 723 who did not receive biologic therapy were included (Table 1). 1:1 propensity‐score matching was performed, resulting in cohorts of 36 330 (Table 1). Over the 10‐year follow‐up period, biologic therapy was associated with a 37% lower hazard of MACE compared with no biologic therapy [2160 vs. 3294; HR: 0.63 (0.60–0.67)] (Table 3).

**TABLE 3:** Risk of developing primary major adverse cardiovascular events in psoriasis patients with and without biologic therapies over a 10‐year period.

Therapy	MACE incidence		Hazard ratio	Risk difference %	p‐value*
	Biologic cohort	Biologic‐naïve cohort			
Any‐biologic (*n* = 36 330)	2160/36 330	3294/36 330	0.63 (0.60, 0.67)	−3.12%	< 0.001
TNFi (*n* = 18 908)	1403/18 908	1844/18 908	0.74 (0.69, 0.80)	−2.33%	< 0.001
IL‐17i (*n* = 5625)	285/5625	450/5625	0.61 (0.53, 0.72)	−2.93%	< 0.001
IL‐23i (*n* = 6387)	238/6387	363/6387	0.64 (0.54, 0.76)	−1.96%	< 0.001
IL‐12/23i (*n* = 3866)	280/3866	374/3866	0.73 (0.62, 0.86)	−2.43%	< 0.001

Abbreviations: Any‐biologic = any FDA‐approved biologics for psoriasis treatment, IL‐17i = interleukin‐17 inhibitor, IL‐23i = interleukin‐23 inhibitor, IL‐12/23i = interleukin‐12/23 inhibitor, MACE = Major Adverse Cardiovascular Events, Non‐White = Black or African American, Asian, American Indian or Alaska Native, Native Hawaiian or Other Pacific Islander, Other Race, TNFi = tumor necrosis factor inhibitor.

* *p*‐values are statistically significant at a threshold of 5%.

When evaluating mutually exclusive biologic class cohorts, each biologic therapy was associated with a lower hazard of MACE compared with biologic‐naïve controls. TNFi use was associated with a 26% lower hazard of MACE over the 10‐year follow‐up period [1403 vs. 1844; HR: 0.74 (0.69–0.80)]. Similarly, IL‐17i use was associated with a 39% lower hazard of MACE [285 vs. 450; HR: 0.61 (0.53–0.72)], IL‐12/23i use was associated with a 27% lower hazard of MACE [280 vs. 374; HR: 0.73 (0.62–0.86)], and IL‐23i use was associated with a 36% lower hazard of MACE over 10 years [238 vs. 363; HR: 0.64 (0.54–0.76)] (Table 3).

### 3.2 MACE Risk by Race

MACE outcomes were further analyzed by race to assess whether the cardiovascular benefits of biologic therapies were consistent across different racial groups. We first compared a pooled Non‐White cohort (Black or African American, Asian, American Indian or Alaska Native, Native Hawaiian or Other Pacific Islander, Other Race) with the White population; then additional analyses separately compared Black and Asian patients with matched White counterparts.

Among patients treated with any biologic therapy, MACE risk did not differ significantly between Non‐White and White populations over 10 years [340 vs. 362; HR: 0.94 (0.80–1.09)]. Similar results were observed when Black and Asian patients were compared separately with White patients (Table 4).

**TABLE 4:** Risk of developing primary major adverse cardiovascular events in psoriasis patients across biologic therapies by race over a 10‐year period.

Therapy	MACE incidence		Hazard ratio	Risk difference %	p‐value*
	**Non‐White cohort**	**White cohort**			
Any‐biologic (*n* = 7138)	340/7138	362/7138	0.94 (0.80, 1.09)	−0.31%	0.394
TNFi (*n* = 2622)	127/2622	162/2622	0.79 (0.62, 0.99)	−1.26%	0.047
IL‐17i (*n* = 971)	52/971	52/971	1 (0.67, 1.49)	0.00%	1.000
IL‐23i (*n* = 946)	32/946	37/946	0.86 (0.53, 1.39)	−0.53%	0.540
IL‐12/23i (*n* = 511)	33/511	30/511	1.11 (0.66, 1.84)	0.59%	0.696
	**Black cohort**	**White cohort**			
Any‐biologic (*n* = 2626)	166/2626	164/2626	1.05 (0.84, 1.31)	0.46%	0.674
TNFi (*n* = 1035)	68/1035	83/1035	0.83 (0.61, 1.15)	−1.45%	0.267
IL‐17i (*n* = 290)	20/290	19/290	0.91 (0.48, 1.70)	0.35%	0.764
IL‐23i (*n* = 321)	11/321	17/321	0.83 (0.30, 1.35)	−1.87%	0.234
IL‐12/23i (*n* = 175)	15/175	14/175	1.04 (0.50, 2.16)	0.57%	0.908
	**Asian cohort**	**White cohort**			
Any‐biologic (*n* = 1946)	76/1946	82/1946	0.87 (0.64, 1.19)	−0.31%	0.383
TNFi (*n* = 599)	30/599	42/599	0.66 (0.41, 1.05)	−2.00%	0.079
IL‐17i (*n* = 313)	12/313	13/313	0.84 (0.38, 1.83)	−0.32%	0.655
IL‐23i (*n* = 300)	10^a^/300	14/300	0.56 (0.24, 1.34)	−1.33%	0.186
IL‐12/23i (*n* = 125)	10^a^/125	10^a^/125	1.08 (0.36, 3.21)	0.00%	0.893

Abbreviations: Any‐Biologic = Any FDA‐approved biologics for psoriasis treatment, IL‐17i = interleukin‐17 inhibitor, IL‐23i = interleukin‐23 inhibitor, IL‐12/23i = interleukin‐12/23 inhibitor, MACE = Major Adverse Cardiovascular Events, Non‐White = Black or African American, Asian, American Indian or Alaska Native, Native Hawaiian or Other Pacific Islander, Other Race, TNFi = tumor necrosis factor inhibitor.

^a^ To ensure patient confidentiality and prevent individual identification, any patient count below 10 is recorded as 10.

* *p*‐values are statistically significant at a threshold of 5%.

When analyzing specific biologic classes by race, Non‐White patients receiving IL‐17i, Il‐23i or Il‐12/23i did not demonstrate a statistically significant difference in MACE risk compared with White patients over the same 10‐year period. In the TNFi‐treated cohort, Non‐White patients had a 21% lower hazard of MACE compared to White populations [127 vs. 162; HR: 0.79 (0.62, 0.99)] (Table 4).

## 4 Discussion

In this study, biologic therapy was associated with a lower 10‐year risk of primary MACE among patients with psoriasis compared with those not receiving biologic treatments. This association was observed across all biologic classes, including TNFi, IL‐17i, IL‐12/23i, and IL‐23i. In race‐stratified analyses, MACE risk was broadly similar across racial groups among biologic‐treated patients, with no consistent signal of increased cardiovascular risk among patients recorded as Non‐White, Black, or Asian compared with White patients. These findings extend prior work by evaluating long‐term MACE risk in a psoriasis‐specific cohort, using mutually exclusive biologic class exposure groups, and examining whether cardiovascular associations with biologic therapy are preserved across racial strata.

Severe psoriasis has been recognized as an independent risk factor for cardiovascular events due to chronic systemic inflammation [4]. Our findings align with previous research indicating that biologic therapies can mitigate the systemic inflammation intrinsic to psoriasis, potentially reducing MACE outcomes. Recent studies provide further context for these findings. Lin et al. used the TriNetX database and reported lower overall cardiovascular disease risk among psoriasis patients who were prescribed biologics versus oral therapies, with reduced risk for TNFi, IL‐17i, and IL‐23i cohorts but not IL‐12/23i [16]. Chen et al. used the TriNetX database and reported no significant risk differences in MACE or venous thromboembolic event in biologic‐naïve psoriatic patients who initiated IL‐17i, IL‐12/23i, or IL‐23i compared to those initiated on TNFi [17]. Song et al. analyzed the Korean national insurance data and observed lower risks of both new‐onset and recurrent MACE with biologics compared with phototherapy [18]. Taken together, these studies and our findings support a favorable cardiovascular profile for biologic therapy in real‐world psoriasis care, while highlighting that the magnitude and class‐specific pattern of benefit may vary according to comparator group, outcome definition, and follow‐up duration.

The present study adds to this literature by placing race stratified cardiovascular outcomes at the center of the analysis. This focus on race stratification is clinically relevant because psoriasis severity, cardiometabolic comorbidity burden, biologic access, and longitudinal specialty care may differ across racial groups [24]. Yet, whether the association between biologic therapy and MACE risk is preserved across racial strata has remained poorly characterized in large, real‐world psoriasis cohorts. By evaluating 10‐year MACE risk in a psoriasis specific population with mutually exclusive biologic class exposure groups, our analysis examines not only whether biologic therapy is associated with lower cardiovascular risk overall, but also whether this association is observed across diverse racial groups. Our findings of broadly consistent MACE risk reduction across biologic classes and racial strata support the cardiovascular safety of biologic therapy in diverse psoriasis populations and underscore the importance of equitable access to effective systemic treatment among patients with substantial cardiometabolic risk. Additionally, our real‐world analysis of multiple biologic classes enriches the existing clinical trial evidence, which has consistently shown that biologics are not associated with elevated cardiovascular events compared to non‐biologic regimens [13, 14, 15].

### 4.1 Racial Aspects in Psoriasis, Biologics, and Primary MACE

The race stratified component of this study should be interpreted within the broader context of known inequities in psoriasis care. Race itself should not be viewed as a biological determinant of cardiovascular response to biologic therapy. Rather, race recorded in electronic health record datasets may reflect overlapping differences in healthcare access, specialist referral, comorbidity screening, cardiovascular risk management, and continuity of care. Considering the well‐established racial disparities in healthcare outcomes [25], it is imperative to examine whether cardiovascular outcomes among biologic treated psoriasis patients differ across racial groups. While real‐world data suggests that response to biologics does not vary between racial/ethnic groups [26], the impact of these treatments on cardiovascular outcomes in different racial groups has not previously been well explored.

Previous investigations have shown that biologics like etanercept have demonstrated consistent safety and efficacy across racial groups [27], though these investigations did not specifically address MACE outcomes. In our analysis, risk was broadly similar across racial groups and most confidence intervals overlapped the null, supporting the interpretation that race was not a major determinant of cardiovascular risk for those on biologic therapy in this dataset. The single nominally significant finding observed in the TNFi Non‐White versus White comparison was modest in magnitude and should be viewed as hypothesis‐generating in light of multiple subgroup comparisons. Several factors may explain this isolated finding. TNFi represented the largest and most established biologic class in our dataset, which may have provided greater statistical power and longer observable exposure than newer classes. Residual differences in access to care, comorbidity burden, treatment adherence, cardiometabolic risk management, or unmeasured psoriasis severity may also have influenced the estimate.

Importantly, this pattern was not consistently reproduced in the Black versus White or Asian versus White race‐specific comparisons, arguing against a definitive race‐specific TNFi effect and favoring cautious interpretation in the setting of multiple subgroup comparisons. Nevertheless, it is important to recognize that psoriasis is less prevalent among Black and Asian populations compared to White populations, yet often presents more severely when it does occur [28, 29]. These distinctions underscore the need for continued assessment of adverse events and outcomes among diverse groups to account for unique clinical presentations and barriers to care. In this context, our findings hold relevance for promoting equitable treatment strategies and reassuring clinicians that biologics can offer comparable cardiovascular benefits across racial backgrounds.

Our study underscores that biologics are associated with a lower incidence of primary MACE in psoriasis patients across racial groups, suggesting that these therapies should be considered a viable treatment option for all patients, regardless of race [30]. This is particularly important as racial minorities often face barriers to advanced treatments like biologics, which may affect disease management and long‐term outcomes [19]. Our findings advocate for equitable access to biologic therapies, especially in underserved populations where systemic healthcare disparities persist. Addressing these disparities could enhance cardiovascular outcomes and improve overall disease management in these populations [20, 31].

## 5 Limitations

Several limitations should be considered when interpreting our findings. First, the observational nature of our study precludes establishing causality. Despite adjusting for numerous confounders, residual confounding may still influence our results. In particular, biologic prescribing is often shaped by healthcare access, insurance coverage, specialist availability, and healthcare utilization patterns, factors that were not fully captured in TriNetX and that may also influence the detection and management of cardiovascular risk. Second, misclassifications of ICD‐10‐CM codes, limited information on psoriasis severity, disease duration, body surface area, and treatment adherence may have affected both exposure and outcome ascertainment. Third, race was derived from structured electronic health record fields, and Unknown Race represented patients for whom race information was not available in the medical record. In addition, the pooled Non‐White category combined heterogeneous racial groups and therefore may have obscured within‐group differences, even though subsequent Black versus White, and Asian versus White comparisons were performed among the largest individually analyzable racial groups in the dataset. Fourth, race‐stratified subgroup analyses involved multiple comparisons; therefore, isolated borderline findings should be interpreted cautiously and viewed as signals requiring confirmation rather than definitive evidence of race‐based treatment effect modification. Fifth, the independently matched control cohorts used for each class‐specific comparison improve internal validity within each comparison but limit direct between‐class comparability of absolute event counts across matched sets. Finally, by excluding patients with exposure to more than one biologic class, the class‐specific analyses prioritized cohort purity at the expense of generalizability to patients who switch biologic mechanisms in routine practice. Consequently, the data should be interpreted as purely observational and hypothesis‐generating.

## 6 Conclusion

Our study demonstrates that biologic therapies are associated with a reduced risk of MACE in patients with psoriasis, with this benefit extending across different biologic classes and racial groups. These findings support the use of biologic therapies for their efficacy in managing psoriasis and mitigating their potential cardiovascular adverse events.

## Funding

This study was supported by the Junior Clinical Investigator Program (JCIP) funded by Sentara Health and Macon and Joan Brock Virginia Health Sciences at Old Dominion University to C.W.E.

## Ethics Statement

IRB exempt.

## Consent

The authors have nothing to report.

## Conflicts of Interest

The authors declare no conflicts of interest.

## Data Availability

The data underlying this article were provided by TriNetX LLC (https://trinetx.com) under the terms of the licensing agreement. Data will be shared on request to the authors, with permission of TriNetX LLC. This study was supported by the Junior Clinical Investigator Program (JCIP) funded by Sentara Health and Macon and Joan Brock Virginia Health Sciences at Old Dominion University to C.W.E.
